# Plasma B-type natriuretic peptide cannot predict treatment response to ibuprofen in preterm infants with patent ductus arteriosus

**DOI:** 10.1038/s41598-020-61291-w

**Published:** 2020-03-10

**Authors:** Seong Hee Oh, Byong Sop Lee, Euiseok Jung, Moon Yeon Oh, Hyun-Jeong Do, Ellen Ai-Rhan Kim, Ki-Soo Kim

**Affiliations:** 1 Department of Pediatrics, Gyeongsang National University School of Medicine, Gyeongsang National University Changwon Hospital, Changwon, Korea; 2 Department of Pediatrics, University of Ulsan College of Medicine, Asan Medical Center, Seoul, Korea

**Keywords:** Predictive markers, Neonatology

## Abstract

Plasma B-type natriuretic peptide (BNP) is a useful marker for diagnosis of hemodynamically significant PDA (hsPDA) and serial BNP measurement is also valuable for monitoring treatment response. This retrospective study was performed to evaluate whether plasma BNP level can predict treatment response to ibuprofen in preterm infants born at <30 weeks of gestation with hsPDA. Plasma BNP was measured before (baseline) and 12 to 24 h after (post-treatment) completion of the first (IBU1) and second (IBU2) course of ibuprofen. We compared the BNP levels of responders (closed or insignificant PDA) with those of non-responders (hsPDA requiring further pharmacologic or surgical closure) to each course of ibuprofen. The treatment response rates for IBU1 (n = 92) and IBU2 (n = 19) were 74% and 26%, respectively. In IBU1, non-responders had lower gestational age and birth weight than responders (both, *P* = 0.004), while in IBU2, non-responders had lower birth weight (*P* = 0.014) and platelet counts (*P* = 0.005) than responders; however, baseline BNP levels did not differ significantly between responders and non-responders in either IBU1 (median 1,434 vs. 1,750 pg/mL) or IBU2 (415 vs. 596 pg/mL). Post-treatment BNP was a useful marker for monitoring treatment efficacy of IBU1 and IBU2 for hsPDA with a cut-off value of 331 pg/mL (*P* < 0.001) and 423 pg/mL(*P* < 0.010), respectively. We did not identify a cut-off baseline BNP level that could predict treatment response to ibuprofen in preterm infants with hsPDA.

## Introduction

Hemodynamically significant patent ductus arteriosus (hsPDA) causes symptoms of congestive heart failure and increases the risk of neonatal morbidities, including necrotizing enterocolitis, intraventricular hemorrhage, acute renal failure, pulmonary hemorrhage, and chronic lung disease, in preterm infants^1–4^. Pharmacologic closure, using a cyclooxygenase (COX) inhibitor, is usually chosen as the first-line treatment for preterm infants with hsPDA. The approximate response rate for the first course of COX inhibitor ranges from 45% to 92%^5–10^. Additional course(s) of COX inhibitor or surgical ligation are considered as second-line treatments in patients who do not respond to the first course of COX inhibitor; however, the closure rates following additional course(s) of COX inhibitors are usually lower than those after the initial course^8–10^. As treatment failure of hsPDA can lead to neonatal morbidities and COX inhibitors are not free from adverse effects, including oliguria and bleeding tendency, neonatologists always face the challenging decision of whether to pharmacologically close or surgically ligate hsPDA, particularly following treatment failure of an initial course of COX inhibitor. In addition, for several echocardiologic findings, laboratory parameters, including plasma prostaglandin, platelet counts, and arterial pH, have been suggested as predictors of hsPDA treatment response^11–13^; however, the utility of these parameters is unclear.

B-type natriuretic peptide (BNP) is a hormone released from cardiomyocytes in response to ventricular volume or pressure overload^14^. Previous studies demonstrated that BNP level correlates well with shunt amount through the ductus, detected by echocardiography, and is a useful marker for diagnosis of hsPDA^15–17^. Serial BNP measurement is also valuable for monitoring treatment response^15,18–20^; however, whether plasma BNP has value as a marker for predicting treatment response to COX inhibitors remains unclear.

The aim of this study was to determine whether baseline plasma BNP levels could predict treatment response to ibuprofen in preterm infants with hsPDA.

## Results

**Figure 1:**
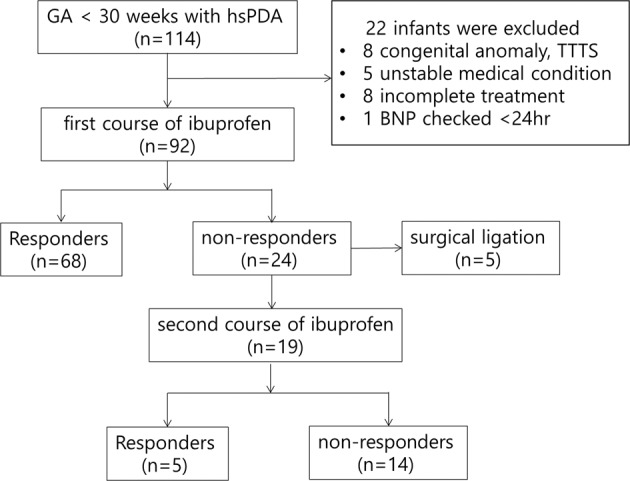
Flowchart of the study population. GA, gestational age; hsPDA, hemodynamically significant patent ductus arteriosus; TTTS, twin-twin transfusion syndrome; BNP, B-type natriuretic peptide.

Among 114 newborns eligible for this study, 22 were excluded based on the exclusion criteria and the remaining 92 were included in the analysis (Fig. 1). Mean gestational age and birth weight were 27.0 ± 1.8 weeks and 939.9 ± 283.8 g, respectively. Baseline characteristics, treatment, and echocardiographic findings are listed in Table 1. Response rates to the first (IBU1, n = 92) and second (IBU2, n = 19) ibuprofen courses were 73.9% (n = 68) and 26.3% (n = 5), respectively. Gestational age and birth weight were greater in responders than non-responders in IBU1, while in IBU2, birth weight, but not gestational age, of responders was greater than that of non-responders. There was greater use of inotropic drugs in responders than non-responders to IBU1. In IBU2, baseline pH and platelet count were lower in non-responders than in responders. The incidence of intravenous ibuprofen therapy and the amount of fluid intake did not differ between the study groups. Echocardiographic parameters that reflect transductal shut volume did not differ between responders and non-responders.

**Table 1:** Clinical characteristics of responders and non-responders to ibuprofen treatment.

	First course of ibuprofen			Second course of ibuprofen		
	Responders (n = 68)	Non-responders (n = 24)	*P*-value	Responders (n = 5)	Non-responders (n = 14)	*P*-value
**Clinical characteristics**						
Gestational age (weeks)	27.7 (23.6–29.9)	26.1 (23.3–29.3)	0.004	26.7 (25.4–28.7)	26.1 (23.9–29.3)	0.754
Birth weight (g)	985 (410–1560)	825 (460–1160)	0.004	1030 (930–1160)	820 (460–1060)	0.014
Male, n (%)	38 (55.9)	14 (58.3)	0.835	4 (80)	7 (50)	0.338
Cesarean section, n (%)	49 (72.1)	14 (58.3)	0.213	3 (60)	10 (71.4)	1.000
Apgar score at 1 min	5.0 (1.0–8.0)	3.5 (1.0–8.0)	0.090	3.0 (2.0–7.0)	4.0 (2.0–8.0)	0.706
Apgar score at 5 min	7.0 (4.0–9.0)	6.0 (2.0–9.0)	0.244	6.0 (2.0–8.0)	7.0 (3.0–9.0)	0.339
Antenatal steroid, n (%)	57 (83.8)	21 (87.5)	1.000	3 (60)	14 (100)	0.058
Chorioamnionitis, n (%)	27 (39.7)	10 (41.7)	0.866	3 (60)	3 (21.4)	0.262
PIH, n (%)	8 (11.8)	6 (25.0)	0.183	1 (20)	4 (28.6)	1.000
Surfactant, n (%)	62 (91.2)	23 (95.8)	0.672	5 (100)	13 (92.9)	1.000
Mechanical ventilation, n (%)	36 (52.9)	15 (62.5)	0.418	4 (80)	10 (71.4)	1.000
Inotropics, n (%)	16 (23.5)	1 (4.2)	0.037	0 (0)	5 (35.7)	0.257
pH	7.27 (7.06–7.46)	7.26 (7.15–7.37)	0.633	7.31 (7.27–7.43)	7.26 (7.15–7.38)	0.034
PaCO_2_ (mmHg)	43.0 (25–78)	46.0 (37–64)	0.072	38.0 (32–56)	45.0 (35–61)	0.186
Creatinine (mg/dL)	1.1 (0.3–2.1)	1.1 (0.5–1.7)	0.968	0.9 (0.5–1.1)	1.0 (0.7–1.9)	0.156
Platelet (×10^6^/mm^3^)	193.5 (97–414)	168.5 (63–359)	0.174	233.0 (131–299)	118.5 (94–217)	0.005
**Treatment characteristics**						
Age at treatment (day)	3.0 (1–15)	2.0 (1–16)	0.244	6.0 (5–25)	6.0 (5–8)	0.391
Intravenous ibuprofen, n (%)	61 (89.7)	20 (83.3)	0.469	4 (80.0)	11 (78.6)	1.000
Fluid intake (mL/kg/d)*	95.5 (53.8–143.7)	97.4 (71.8–138.4)	0.099	105.6 (92.4–119.5)	89.5 (78.2–116.0)	0.070
Urine output (mL/kg/h)*	2.8 (0.8–5.1)	2.5 (1.3–4.8)	0.895	2.3 (1.4–2.8)	2.0 (1.5–3.6)	0.622
**Echocardiographic characteristics**						
Ductus diameter (mm)	2.4 (1.5–5.3)	2.2 (1.4–3.5)	0.128	2.7 (1.7–3.8)	2.1 (1.4–2.5)	0.186
Ductus diameter to body weight (mm/kg)	2.4 (1.2–5.6)	2.7 (1.9–4.9)	0.102	2.6 (1.8–3.3)	2.5 (1.9–4.3)	0.687
LA/Ao ratio	1.7 (1.0–2.8)	1.7 (1.0–2.8)	0.985	1.8 (1.4–2.0)	1.8 (1.2–2.8)	0.574
Transductal peak systolic velocity (m/s)	1.6 (0.6–3.9)	1.3 (1.0–3.9)	0.738	1.4 (1.0–2.0)	1.1 (0.8–2.1)	0.776
Large transductal shunt volume, n (%)^†^	38 (55.9)	9 (37.5)	0.121	3 (60)	12 (85.7)	0.272

PIH, pregnancy-induced hypertension; LA/Ao, left atrium/aorta.

Data are presented as median (range) or number (%).

*Fluid intake and urine output are average data measured during the days of ibuprofen therapy.

^†^A large transductal shunt volume was defined by the presence of one of the following parameters: (1) absolute ductus diameter (mm) ≥2.0; (2) ductus diameter indexed to body weight (mm/kg) ≥1.4, (3) transductal peak systolic velocity (m/s) <1.5; and (4) LA/Ao ratio >2.0. No patients were categorized into the group of small shunt volume.

Among the echocardiographic parameters available, the ductal diameter indexed to body weight and the left atrium to aorta (LA/Ao) ratio were positively correlated with baseline BNP level of IBU1 (Table 2). The median (range) baseline BNP levels were significantly greater in the patients with large shunt volume (2,167 [82–5,000] pg/mL) than those with moderate shunt volume (1,000 [105–5,000] pg/mL) (*P* = 0.043) in IBU1. There was no correlation between the echocardiologic parameters and the baseline BNP levels in IBU2.

**Table 2:** Correlation between the baseline BNP levels and the echocardiographic parameters in the study population.

	Baseline BNP in IBU1 (n = 92)		Baseline BNP in IBU2 (n = 19)	
	R	*P*-value*	R	*P-*value*
Ductus diameter (mm)	0.152	0.147	0.294	0.222
Ductus diameter to body weight (mm/kg)	0.337	0.001	0.315	0.188
LA/Ao ratio	0.212	0.049	0.205	0.416
Transductal peak velocity	−0.093	0.626	−0.124	0.842

^*^Pearson’s correlation coefficient (for IBU1) or Spearman’s rank correlation test (for IBU2).

**Figure 2:**
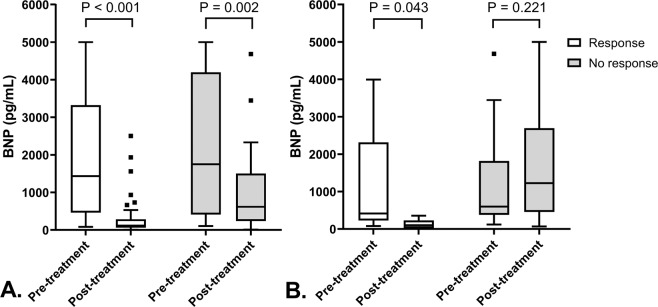
Box plots of plasma BNP levels of responders and non-responders before and after the first (**A**) and second (**B**) courses of ibuprofen treatment. Closed rectangles represent outliers, with BNP levels more than 1.5 times the interquartile range (IQR).

Baseline BNP levels did not differ between IBU1 or IBU2 responders and non-responders (Table 3); however, responders had significantly lower post-treatment BNP levels than non-responders after both courses of ibuprofen. Following IBU1, BNP levels decreased significantly in both responders (*P* < 0.001) and non-responders (*P* = 0.002), whereas, after IBU2, BNP levels decreased significantly in responders (*P* = 0.043), but not in non-responders (*P* = 0.221) (Fig. 2).

**Table 3:** Changes in BNP levels in responders and non-responders to the first and the second courses of ibuprofen.

	First course of ibuprofen			Second course of ibuprofen		
	Responders (n = 68)	Non-responders (n = 24)	*P*-value	Responders (n = 5)	Non-responders (n = 14)	*P*-value
Pre-BNP (pg/mL)	1,434 (82–5,000)	1,750 (105–5,000)	0.796	415 (77–3,995)	596 (121–4,684)	0.622
Post-BNP (pg/mL)	119 (6–2,504)	616 (11–4,684)	<0.001	101 (16–355)	1,225 (66–5,000)	0.007
Post- to pre-BNP ratio (%)^*^	8 (1–147)	31 (6–1012)	<0.001	16 (3–86)	105 (18–840)	0.005

BNP, B-type natriuretic peptide. Values are presented as median (range).

*(Post-BNP/pre-BNP) × 100.

**Figure 3:**
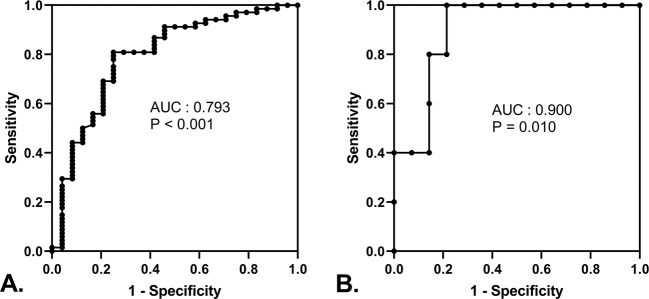
ROC curves describing the post-treatment BNP cut-off values predicting persistent hsPDA after first (**A**) and second (**B**) courses of ibuprofen treatment. Area under the curve values after the first and second courses were 0.793 (95% CI, 0.681–0.904; *P* < 0.001) and 0.900 (95% CI, 0.759–1.000, *P* = 0.010), respectively.

We performed a receiver operator characteristic (ROC) curve analysis, which revealed a significant association between BNP level and persistent hsPDA after each course of ibuprofen (Fig. 3). The cut-off post-treatment BNP levels that reflected persistent hsPDA after IBU1 and IBU2 were 331 pg/mL (sensitivity: 75.0%, specificity: 80.9%) and 423 pg/mL (sensitivity: 78.6%, specificity: 100%), respectively.

Among the patients who did not respond to IBU1 (n = 19) and were subsequently subjected to IBU2, neither baseline nor post-treatment BNP levels measured for the previous ibuprofen course (i.e., IBU1) could predict response rate to IBU2 (Table 4).

**Table 4:** Pre- and post-BNP levels of the previous (first) course of ibuprofen in responders and non-responders to the second course of ibuprofen.

	Responders (n = 5)	Non-responders (n = 14)	*P*-value
Pre- BNP (pg/mL)	908 (151–2,749)	3,842 (180–5,000)	0.087
Post- BNP (pg/mL)	355 (77–648)	749 (104–4,684)	0.087
Post- to pre-BNP ratio (%)^*^	25 (9–235)	31 (6–212)	0.893

BNP, B-type natriuretic peptide. Values are presented as median (range).

*(Post-BNP/pre-BNP) × 100.

## Discussion

As previously shown^15,20^, BNP levels of responders to ibuprofen therapy significantly decreased after each course of treatment and thus clearly reflected treatment response, with good sensitivity and specificity. The baseline BNP levels obtained prior to IBU1 were also associated with the transductal shunt volume defined by several echocardiographic parameters including LA/Ao ratio^15–17^; however, baseline BNP levels, measured before either IBU1 or IBU2, could not predict treatment response in preterm infants with hsPDA.

To the best of the authors’ knowledge, there has only been one previous study of the role of BNP as a predictive marker for response to COX inhibitor therapy against PDA. In contrast to our findings, Hsu *et al*. reported that baseline BNP levels in preterm infants with large PDA were higher in non-responders than responders to indomethacin therapy^18^. They suggested a cut-off baseline BNP level of >1,805 pg/mL, with relatively high sensitivity and specificity, based on data from a smaller number of patients (n = 31) than that included in the present study. The reason for this discrepancy is unclear. Several antenatal and postnatal factors have been suggested as parameters for predicting poor responsiveness, including gestational age, birth weight, pregnancy-induced hypertension, use of antenatal steroids, respiratory distress syndrome, small for gestational age, and postnatal age at the first pharmacologic treatment^21–23^. In the present study, gestational age was lower and the use of antenatal steroids and surfactant appeared to be greater than in the study reported by Hsu *et al*.^18^.

In the present study, the transductal shunt volume defined by several echocardiologic parameters including ductal diameter and LA/Ao ratio were not predictive of ibuprofen response. Although these parameters are regarded as good markers for the severity and natural course of PDA^15,20,24^, studies of the echocardiographic parameters that predict drug response rate are scarce. In a small study of preterm infants <28 weeks of gestation, the baseline ductal diameter did not differ between responders and non-responders to the first ibuprofen course^25^. By contrast, in a study of relatively mature preterm infants (gestational age <35 weeks), an index of PDA diameter squared/birth weight >9 mm^2^/kg was reported to predict failure of treatment with indomethacin^26^. Similarly, in a multicenter cohort study, ductal diameter >2 mm was suggested as an indicator of ibuprofen treatment response; however, the specificity (0.44) and positive predictive value (0.29) of ductal diameter alone were insufficient^27^. Ductal flow velocity, which reflects higher pulmonary arterial pressure, was suggested as a good predictive marker of response to COX inhibitors in a study of infants of 22–27 weeks’ gestation^28^. Hsu *et al*. reported that neither ductal diameter nor LA/Ao ratio was a predictive factor for responsiveness to COX inhibitors^18^, similar to our findings.

The second course of COX inhibitors usually has a lower treatment response rate than the first course^8–10^, and may harbor a higher risk of complications without therapeutic benefit^5,8,10^. We speculated that the degree of change in BNP level before and after IBU1, which may reflect the individual response pattern of ductus to ibuprofen, could predict subsequent treatment response to the same drug. Unfortunately, although both first course pre- and post-treatment BNP levels tended to be higher in non-responders than responders to IBU2, the difference did not reach statistical significance. There has been a limited number of studies regarding predictors of treatment outcomes following a second course of COX inhibitors. Louis *et al*. suggested that advanced gestational age was a predictor of non-response to a second course of indomethacin therapy^29^. Another retrospective study also reported that persistent ductal Doppler flow within 24 h of completed initial indomethacin course was a significant predictor of failure of the second course^30^; however, the study did not provide any quantitative data for assessment of ductal shunt flow.

In IBU2, baseline platelet counts were lower in non-responders than in responders. Whether platelet adhesion plays a crucial role in the closure of ductus arteriosus remains controversial, especially in preterm infants^31^. In two recent meta-analyses, a significant relationship was detected between low platelet count in the early days of life and incidence or treatment failure rate of primary COX inhibitors for hsPDA^32,33^. Another recent large study reached a similar conclusion to the present study; platelet counts after the first course of COX inhibitors and before and after the second course were lower in non-responders than in responders, although initial baseline platelet counts did not differ between the groups^34^. The incidence of pregnancy-induced hypertension did not differ between responders and non-responders.

There are several limitations to this study. First, the sample size, particularly the number of patients who underwent IBU2, was insufficient to address the predictive value of baseline BNP levels. This may have simply resulted in type 2 statistical error, although our population was larger and less heterogenous (in terms of gestational age) than that described in the study of Hsu *et al*.^18^. Second, the decision of whether or not to administer additional ibuprofen or surgically ligate the ductus may have been influenced by baseline BNP levels. That is, surgical closure is more likely to be preferred than ibuprofen treatment in patients with very high baseline BNP levels, which could lead to selection bias for treatment response to the second course of ibuprofen. Third, our retrospective study lacks a well-designed echocardiographic protocol for the assessment of hemodynamic status such as shunt volume. However, quantitation of transductal shunt flow is not always feasible and can only be estimated based on several echocardiographic parameters. We tried to provide the categorized comparative data on the shunt flow by using several echocardiographic indices available. As previously depicted, no difference in the transductal shunt volume was found between the responders and non-responders to both IBU1 and IBU2.

In conclusion, we could not determine a cut-off baseline BNP level able to predict treatment response to ibuprofen in preterm infants with hsPDA. Plasma BNP levels correlated well with PDA hemodynamic status; however, their role as a guide to assist neonatologists in choice of therapeutic modality, particularly for non-responders to an initial standard COX inhibitor course, is questionable. To develop guidelines for optimal treatment for hsPDA, well-designed studies, considering various risk factors that affect ductal patency and response to COX inhibitors, are warranted.

## Methods

The retrospective review of the medical records of patients hospitalized in the neonatal intensive care unit in Asan Medical Center, Seoul, Korea from 2010 to 2016 was performed. During the study period, data on echocardiography and plasma BNP levels were collected, according to our local protocol for management of hsPDA. The clinical characteristics and BNP levels of responders and non-responders to the IBU1 and IBU2 were compared (see below). The study was approved by the institutional review board of Asan Medical Center (IRB No. 2017–0253), who granted a waiver of informed consent. All methods were performed in accordance with the relevant guidelines and regulations.

### Subjects

Eligibility criteria for inclusion of newborn infants in this study were as follows: (1) born at gestational age <30 weeks; (2) diagnosis of hsPDA meeting clinical and echocardiographic criteria (see below); (3) treated with oral and/or intravenous ibuprofen after 24 h of age; and (4) available echocardiography and plasma BNP data before (baseline) and after (post-treatment) each course of ibuprofen treatment. Exclusion criteria were newborns with the following: (1) major congenital anomalies, including congenital heart disease and twin to twin transfusion; (2) unstable medical conditions during the time of ibuprofen treatment that may have affected cardiac function, such as pneumothorax, pleural effusion, and septic shock; (3) incomplete initial course of ibuprofen (<3 doses), due to presentation of ibuprofen contraindications such as oliguria, necrotizing enterocolitis, bowel perforation, or death; or (4) baseline BNP level obtained <24 h after birth.

### Diagnostic criteria for hsPDA

A hsPDA was diagnosed when an infant fulfilled both of the following clinical and echocardiographic criteria. The clinical criteria were met if the infant had two or more of the following symptoms: (1) systolic or continuous cardiac murmur at the left sternal border; (2) precordial impulse, bounding pulse, or hypotension; (3) respiratory symptoms, such as tachypnea, apnea, increased PaCO_2_, or increased ventilator needs; and (4) increased pulmonary vascular marking or cardiomegaly on chest radiography. Echocardiographic criteria were as follows: (1) ductal diameter ≥ 1.5 mm with left to right shunt;^35,36^ and/or (2) LA/Ao ratio ≥ 1.4^36^. Echocardiography was performed by attending neonatologists with a Philips IE33 (Philips Ultrasound, Bothell, WA, USA) before (baseline) and between 12 and 24 h after (post-treatment) each course of ibuprofen, or as otherwise indicated. A two-dimensional color flow Doppler was performed to check shunt at the ductus arteriosus. Ductal diameter and transductal peak systolic velocity and systolic-to-diastolic velocity gradient was measured in the parasternal short axis, and LA/Ao ratio was measured using M-mode in the parasternal long axis. To address the impact of hemodynamic status on the ibuprofen responsiveness, transductal shunt volume was categorized into moderate or large according to the classification suggested by de Boode *et al*.^37^ A large transductal shunt volume was defined by the presence of at least 2 of the following parameters: (1) absolute ductal diameter (mm) ≥ 2.0; (2) ductal diameter per body weight (mm/kg) ≥ 1.4; (3) transductal peak systolic velocity (m/s) <1.5; and (4) LA/Ao ratio >2.0.

### Measurement of plasma BNP

According to our local protocol, bedside echocardiography and plasma BNP measurement were indicated for every infant who met the clinical criteria for hsPDA and/or were treated with ibuprofen. Plasma BNP levels were measured at least twice per patient, i.e., pre- (baseline) and post- (between 12 and 24 h after the last ibuprofen dose of each course) ibuprofen treatment. Blood samples were immediately placed in EDTA-treated tubes, transported to the laboratory department of our hospital, and analyzed by sandwich fluorescence immunoassay (Siemens Healthineers, NY, USA). The measurable range of the BNP assay was 2–5,000 pg/mL. BNP values reported as >5,000 pg/mL by our laboratory were counted as 5,000 pg/mL for this study.

### Treatment with ibuprofen

One course of intravenous or oral ibuprofen treatment comprised three doses administered at intervals of 24 h. The doses of ibuprofen were 10 mg/kg/day for the first day, and 5 mg/kg/day for the second and the third days. In the patients. The PDA still fulfilled hsPDA criteria after completion of IBU1, an additional ibuprofen course was started, unless contraindicated. When the PDA was open but did not meet the criteria for hsPDA, follow-up echocardiography was performed 12–24 h later, without an additional course of ibuprofen. PDA ligation was performed when hsPDA remained after two (or three) courses of ibuprofen, or when ibuprofen was contraindicated.

### Definition of response to ibuprofen

Patients who completed each course of ibuprofen treatment were categorized as “responders” or “non-responders”. “Responders” were defined as infants with (1) closed PDA, or (2) a small PDA that did not meet the diagnostic criteria for hsPDA and thus did not need any further pharmacologic or surgical treatment for PDA within 1 week after previous treatment. “Non-responders” were defined as infants who met the criteria for hsPDA after ibuprofen treatment, and were treated with an additional course of ibuprofen and/or surgical ligation.

### Statistical analysis

Statistical analyses were performed using SPSS version 24.0 for windows (SPSS Inc., Chicago, IL, USA). Continuous variables were evaluated using the Student’s t test or Mann-Whitney U test. Categorical variables were tested by chi-square or Fisher’s exact test. The correlations between baseline BNP levels and the echocardiographic parameters were determined using Pearson’s correlation coefficient or Spearman’s rank correlation test. Changes of BNP levels from baseline to post-treatment were evaluated using the Wilcoxon signed rank test. ROC curve analysis was performed to determine the cut-off BNP level for detection of PDA closure. *P* < 0.05 was considered statistically significant.
